# Gradient porous structures of mycelium: a quantitative structure–mechanical property analysis

**DOI:** 10.1038/s41598-023-45842-5

**Published:** 2023-11-07

**Authors:** Eric Olivero, Elzbieta Gawronska, Praveena Manimuda, Devyani Jivani, Faemia Zullfikar Chaggan, Zachary Corey, Thaicia Stona de Almeida, Jessica Kaplan-Bie, Gavin McIntyre, Olga Wodo, Prathima C. Nalam

**Affiliations:** 1 Department of Materials Design and Innovation, University at Buffalo, Buffalo, NY 14226 USA; 2 Faculty of Mechanical Engineering and Computer Science, Czestochowa University of Technology, 42201 Czestochowa, Poland; 3 Horiba Instruments Inc, Piscataway, NJ 08854 USA; 4 Ecovative Design LLC, 60 Cohoes Ave, Green Island, NY 12183 USA

**Keywords:** Engineering, Materials science, Mathematics and computing

## Abstract

Gradient porous structures (GPS) are characterized by structural variations along a specific direction, leading to enhanced mechanical and functional properties compared to homogeneous structures. This study explores the potential of mycelium, the root part of a fungus, as a biomaterial for generating GPS. During the intentional growth of mycelium, the filamentous network undergoes structural changes as the hyphae grow away from the feed substrate. Through microstructural analysis of sections obtained from the mycelium tissue, systematic variations in fiber characteristics (such as fiber radii distribution, crosslink density, network density, segment length) and pore characteristics (including pore size, number, porosity) are observed. Furthermore, the mesoscale mechanical moduli of the mycelium networks exhibit a gradual variation in local elastic modulus, with a significant change of approximately 50% across a 30 mm thick mycelium tissue. The structure-property analysis reveals a direct correlation between the local mechanical moduli and the network crosslink density of the mycelium. This study presents the potential of controlling growth conditions to generate mycelium-based GPS with desired functional properties. This approach, which is both sustainable and economically viable, expands the applications of mycelium-based GPS to include filtration membranes, bio-scaffolds, tissue regeneration platforms, and more.

## Introduction

Gradient porous structures (GPS) are commonly found in natural tissues and biological matter, exhibiting transitions in microstructure, biochemical composition, and mechanical properties along a specific axis^1,2^. The natural GPS possesses remarkable functional properties, such as high load-bearing capacity and superlubricity in knee cartilage^3,4^, improved stiffness in bones and eggshells, flexibility and semi-permeability in the skin, among others.

Engineered GPS hold significant technological potential in tissue engineering, filtration technologies, cultured meat production, functional coatings, and other applications^5–11^. Existing synthetic approaches for generating GPS involve methods such as diffusion-based polymerization^12^, multilayer electospinning^13^, microfluidic mixing^14^, and additive manufacturing technologies^15^, among others^16^. However, these approaches often entail complex, multi-step processes and require additional functionalization steps to achieve scaffolds with desired properties. Furthermore, scalability remains a challenge for many of these methods. Therefore, this study explores the potential of mycelium, the root structure of fungi, as an economically viable and self-growing fibrous biomaterial with the ability to generate gradient porous structures.

Mycelium, the three-dimensional network of mushroom hyphae (filamentous fibers composed of elongated cells), naturally grows in the soil. The cell walls of hyphae contain various biopolymers, including lipids, chitin (a polysaccharide that contributes to rigidity), β-glucans, and other glycoproteins^17^. These components provide inherent stiffness to the network and offer a wide range of surface-active chemical groups. Mycelium has recently gained attention as a programmable matter, where the structural features of the network can be controlled by manipulating growth conditions, species type, feed substrate composition, and other factors^18–22^. Numerous studies have shown that physical and nutritional variations in the environment where the mycelium grows influence the growth direction and anastomosis (bifurcation or fusion) of leading hyphae^23,24^. These factors, in turn, affect the local network connectivity and architecture. The biomanufacturing of mycelium-based materials, which involves baking and drying the mycelium structures, is already emerging as a sustainable alternative material for packaging, textiles, acoustic paneling, and architectural components^23,24^. The programmability of mycelium structure during production enables the development of porous structural materials with desired properties, holding potential for various unexplored applications. In recent developments, mycelium has shown promise as a bio-scaffold for tissue engineering. Autoclaved mycelium networks have exhibited biocompatibility with primary human dermal fibroblasts, enabling their direct attachment and proliferation within the network^25^.

Mycelial fibers grow to form porous networks when cultivated intentionally (dominant vertical growth) in trays containing agricultural waste and inoculates. Similar to other biological fibrillary networks like wood hemicellulose^20^ and collagen^21^, mycelium exhibits a strong correlation between its structural characteristics and bulk mechanical properties. In a study by Haneef et al*.*, the addition of potato dextrose broth (PDB) to the feeding substrate was found to stimulate the biosynthesis of plasticizers (lipids and proteins) while reducing the chitin content in the hyphal wall of mycelium. As a result, the elastic modulus of the mycelium network decreased by approximately 2 to 3 times (and exhibited a ~ 130% increase in elongation) compared to the network grown without PDB^22^. Switching the mycelium species also demonstrated changes in the hyphal chemical composition, which impacted the elastic modulus of the mycelium network^22,26^. Furthermore, environmental growth conditions such as CO_2_ levels, temperature, moisture, and light conditions directly influence the density of the mycelium network (ranging from 0.029 to 0.35 g/cm^3^), leading to variations in mechanical moduli (ranging from 0.6 to 100 MPa)^27–29^. When analyzing the mechanical properties of mycelium networks using the Ashby plot, they exhibit behavior similar to foams or other random polymer networks^27,29^. The macroscale compression measurements by Islam et al*.* on a similar dried fibrous mycelium showed an open cell foam response with linear elastic deformation at small compressive strains (< 0.1), followed by a plateau regime at higher strains^27^. A multiscale continuum model accounting for local density fluctuations in the mycelium tissue enabled to address the observed strain hardening behavior of the mycelium network under axial compressive and tensile loading.

Previous studies on mycelium networks conducted macro-mechanical testing, assuming uniform structural features throughout the network. However, as mycelial growth progresses, the younger and newly formed hyphal branches extend farther away from the inoculant source and feed material, potentially leading to variations in network morphology and structural connectivity^30^. The detailed understanding of how the structural features of mycelium vary with growth direction and their impact on mechanical properties still remains unclear. This paper proposes that gradient porous structures are generated as the mycelium network grows vertically. To investigate this phenomenon, we conduct a comprehensive microstructural characterization of the mycelium network and establish correlations between its structural features and the local elastic moduli. The mycelium tissue is carefully sectioned along the growth direction, and high-resolution scanning electron microscope (SEM) images are obtained to analyze the mycelium network. We utilize microstructure informatics tools to extract morphological and topological characteristics of the random fibrous networks, including hyphal radius, average segment length, crosslink density, network density, porosity, and pore area. Additionally, for the first time, we employ a soft micro-indentation technique to estimate the meso-scale mechanical moduli of the mycelium network and investigate how they vary with the distance from the feed substrate. Our findings reveal a subtle yet noticeable change in both the structural characteristics and elastic modulus of the mycelium network. This study suggests that by harnessing the inherent property of hyphal growth and its sensitivity to the surrounding environment and food sources, it is possible to develop a cost-effective mycelium-based GPS with texture-appropriate properties.

## Materials and methods

### Sample preparation

**Figure 1:**
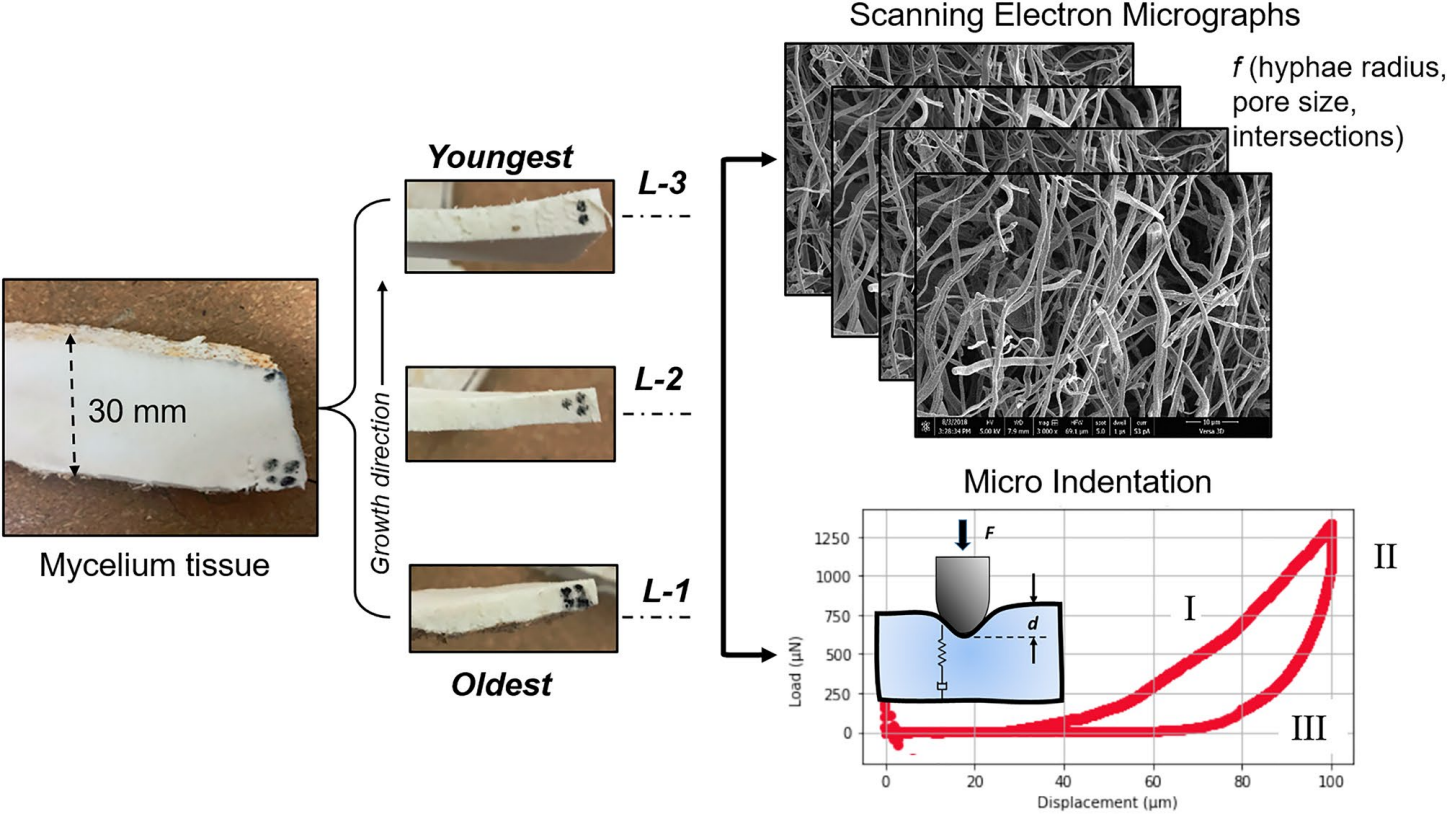
The mycelium tissue (dried) is sectioned along the growth direction, and each mycelium section is represented as L-1, L-2, and L-3, with L-1 representing the oldest growth (closest to the feed substrate), and L-3, the youngest growth of the mycelium tissue. Representative scanning electron micrographs of the hyphal network for microstructural extraction (right, top) and a representative force-indentation depth (*F-d*) curve (right, bottom) measured on mycelium tissue are presented. Three stages in the *F-d* curve, with stage I representing the loading curve, stage II the dwell at a constant depth, and stage III the unloading curve until the probe is removed out of contact, are shown.

Dried mycelium tissues, obtained from Ecovative Design LLC, were used as received for the experiments. To cultivate the mycelium, agricultural feed along with mycelium inoculates were placed into trays, and the hyphal network was allowed to grow in bioreactors for approximately nine days under controlled growth conditions. The mycelium tissue typically reached a thickness of around 30 mm during this growth period. After the growth process, the fungal tissue was dried at an elevated temperature of 82 °C until it reached an equilibration moisture content, preserving the structure and halting further growth. To conduct growth-dependent studies, the mycelium was sectioned along its thickness using a microtome, as depicted in Fig. 1. The layer closest to the feed substrate, representing the oldest growth, was labeled as L-1, while the subsequent layers along the growth direction were designated as L-2 and L-3, respectively. The top layer of the tissue, which was exposed to environmental factors, was removed from consideration and not included in the study. Each layer with an approximate thickness of 8.9 mm was (a) imaged and characterized to assess microstructural features and (b) micro-indented to estimate local mechanical moduli.

### Microstructural imaging

The structural features of the fibrous network were extracted from the high-resolution SEM images (Hitachi S4000, FESEM). The sliced fungal sections were coated with a thin conducting layer of gold-platinum to minimize charging effects during imaging. The SEM images were collected from the three mycelium sections (*i.e.*, L-1, L-2, and L-3) at various magnifications, including 800×, 1kX, 3kX, and 15kX. A minimum of five images per magnification were taken from different locations on the sample to ensure statistical relevance. While SEM images at 15kX magnification provided an accurate estimation of individual fiber radius, the limited number of fibers per image made it challenging to extract statistically meaningful values for other microstructural descriptors such as pore size, porosity, and crosslink density. On the other hand, low magnification images were constrained by resolution limitations, where fibers with radii smaller than 0.2 µm were represented by only a few pixels per diameter, reaching the resolution limit of the image. The influence of image magnification on data resolution is discussed in detail in supplementary information S1. Consequently, SEM images acquired at a resolution of 3kX were chosen to quantify the microstructural characteristics (see Fig. 1).

### Micromechanical testing

In this study, the linear elastic modulus of the mycelium is measured by performing microindentation (compressive) measurements using Hysitron Biosoft™ In situ indenter (Bruker Instruments, Minneapolis, USA). The linear-elastic deformation of the porous network assumes a homogeneous density of the solid component and the microscopic elastic (Young’s) modulus of the network. The microscopic elastic modulus corresponds to the minimized elastic energy stored at the individual nodes under applied strain. The micron-size indenter with small indentation depths enables the estimation of the local elastic properties of the network.

BioSoft™ In-situ indenter enables non-destructive multiscale mechanical testing of soft and biological materials, offering high precision in force sensitivity (< 1 nN) and a wide displacement range of up to 150 μm. The sectioned and dried mycelium tissue was glued to a glass substrate to ensure no-slip conditions during the indentation measurements. Displacement-controlled indentations were performed on all mycelium sections. The force-indentation depth (*F-d*) curves were acquired using a diamond cone indenter with a radius (*R*) of 92.03 μm. Figure 1 displays a representative *F-d* curve obtained on the dried mycelium tissue. The *F-d* curve presents three stages: stage I corresponds to the loading process with a loading rate of 3 µm/s. Once the maximum (set) displacement was reached, the indenter was held at a constant indentation depth for 50 s (Dwell) before the stress was released (Stage II). In stage III, the indenter was retracted at an unloading rate of 1 µm/s until the indenter was pulled entirely out of contact with the sample. Calibration of machine compliance was conducted on a reference sample quartz. A minimum of five to six indents were made per mycelium section, with sufficient spacing between the indents. The measurements were conducted on two different mycelium tissues grown under similar conditions. The *F-d* curves were analyzed using IGRO 6.30 Software.

## Microstructural analysis of heterogeneous mycelium network

The analysis consists of three steps: (i) the segmentation of micrographs to partition the image into regions corresponding to hyphae and background, (ii) the processing of the segmented image to extract the descriptors, and (iii) the analysis of each descriptor as a function of growth direction. Multiple segmentation algorithms are employed on the same micrograph to capture the unique characteristics of the targeted features. The approach is necessary due to the heterogeneous nature of the fibrous mycelium, which presents challenges in extracting microstructural parameters. These challenges include difficulties in automatically distinguishing fiber edges for partially overlapping fibers, detection of partial (cut out by the edges of the micrographs) and fake (inaccurate identification) pores, and misidentifying intersection points for multi-fiber overlap or hyphae splitting.

To address these challenges, SEM micrographs are analyzed using ImageJ software (version 1.52a) with a combination of semi-automated algorithms and manual annotation. The open-source tool DiameterJ (version 1-018) is employed to analyze the morphology of the hyphal network^31^. DiameterJ facilitates automated SEM micrograph analysis to quantify various features of the fibrous network, including fiber diameter, pore size, porosity, and fiber crosslink density. In cases where the selected segmentation algorithm in DiameterJ was sensitive to the selected segmentation algorithm^32^, alternative approaches are adopted and detailed in the study.

*Fiber radii distribution*: Raw images are segmented and processed using DiameterJ as described in the supporting information S2. From the segmented images, (a) the distance transformation matrix, *i.e.,* the Euclidean distance for each pixel in the fiber to its nearest orthogonal background pixel, and (b) the central line of the skeleton by recursively eroding the edges of the fibers are extracted. Combining the two transformations allows for the determination of the fiber radius—the distance from each centerline pixel to its nearest interface with the orthogonal pore. The radii cannot be accurately measured at the intersection between two fibers as the distance to the nearest interface no longer represents the fiber diameter. DiameterJ excludes the intersection region for the radii calculation and generates a distributed histogram of fiber radii.

Based on the histogram analysis, we observed a few dominant types of hyphae with varying diameters within the network. To accurately quantify and separate these different fiber types, Gaussian Mixture Model (GMM) was employed. The GMM allowed the deconvolution of the contributions of each fiber type to the histogram by identifying multiple distinct Gaussian distributions. Each distribution represents a dominant fiber type characterized by its mean diameter, variance, and corresponding mixing weight. The number of dominating fiber types is predetermined using Akaike information criteria (AIC), which prevents overfitting the fiber radius histogram. A few representative images (extracted from each section), along with AIC and GMM fits, are shown in the supporting information S3. To validate the accuracy of our methodology, we compared the fiber radii values estimated by the GMM procedure with discrete diameter measurements obtained from individual fibers in a few representative images. The comparison demonstrated an error of less than 10%, indicating the robustness and reliability of the methodology employed in accurately capturing the fiber characteristics within the mycelium network.

*Pore size distribution*: To accurately extract the parameters related to the pores formed by the top fibers, the micrographs were manually segmented. This approach minimized the contribution of fibers present underneath the top few layers that could potentially introduce errors in determining pore features. Specifically, the three topmost layers of fibers visible in the SEM micrographs were employed in the manual segmentation. This approach also eliminated partial pores at the edge of the micrograph and prevented the inclusion of fake pores that may be formed by fibers present in the deep background, ensuring the inclusion of only complete pores and capturing mostly those present in the central part of the micrograph. DiameterJ was employed on the manually segmented images to extract pore areas and further processed to construct the corresponding histograms of pore areas.

*Network density and segment length*: The segmentation employed for pore size estimation was used to determine network characteristics. To compute network characteristics, the skeleton of the mycelium was determined using the classic skeletonization algorithm^33^ and then segmented into individual segments constituting the skeleton^34,35^. Once obtaining the network skeleton, two network characteristics: (a) the *network density* representing the total length of the skeleton connected to the network per unit of network volume and (b) the *segment length* referred as the average length of the branches between two intersections along the skeleton, were computed. Similar to pore area calculations, the incomplete branches/segments at the boundary of the micrograph were excluded. Further, only segments within the range of 1–10 μm were considered for accurate calculations of the average segment length. This range was chosen to exclude very short segments (typically artifacts) with a length of only a few pixels (< 1 μm) and long segments (> 10 μm) that were found to be non-representative of the overall microstructure.

*Crosslink density*: The segmentation process for estimating the crosslink density in the mycelium network involved careful consideration of fiber overlap and bundling. Manual annotation using an e-tablet was employed, focusing on capturing the central axis of hyphae with intersections rather than precise skeleton representation. Special attention was given to accurately annotate the overlapping hyphae, which can be challenging with other segmentation methods. The resulting skeleton was used to determine the number of intersections and compute the crosslink density, representing the number of hyphae intersections per unit area of the micrograph. The slight deviation in skeleton location did not affect the accuracy of the analysis, as the exact skeleton position was not critical for the study.

*Network porosity*: The percentage porosity of the network was estimated using DiameterJ, defined as the ratio of the total pore area to the micrograph area. The segmentation employed for fiber radius analysis was employed for porosity quantification.

Although different segmentation methods were employed in this study, the tailored procedures allowed for accurate extraction of the target microstructural features for the random fibrous networks and avoided the artifacts usually generated when a single image segmentation and processing approach is employed. Further, at least 3–5 participants were involved in analyzing structural parameters to reduce the error involved in developing or selecting the segmentation, which helped improve the accuracy of the parameter analysis.

## Results and discussion

### Growth-dependent microstructural characterization of intentionally grown mycelium

**Figure 2:**
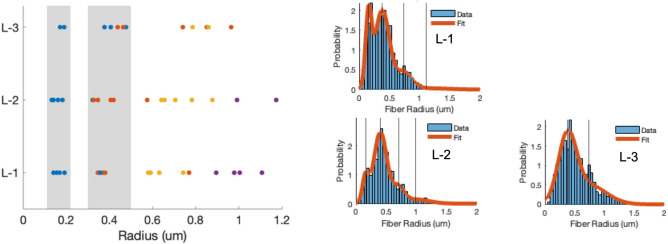
The average radii (*r*, in μm) of the dominating hyphal fibers (estimated from GMM) are shown as a function of growth depth (L-1, L-2, L-3). Dominant radii are color-coded with four colors (blue, orange, yellow, and purple) to represent the order of the extracted means from the histograms. The two thinnest types of hyphae (marked blue and orange) are additionally highlighted with a grey background. The representative histograms of fiber radius extracted from SEM micrographs acquired at L-1, L-2, and L-3 and the corresponding GMM fits (orange line) are shown on the right panels.

*Hyphal Radius (r)*: The right panels in Fig. 2 depict representative histograms of hyphal radii extracted from SEM micrographs at different depths of mycelium tissue, i.e., L-1, L-2, and L-3. The GMM fits to the histograms (depicted as the orange line) identify the dominant fiber types present in each layer. The analysis indicates that mycelium from the younger layer (L-3) predominantly consists of two to three distinct fiber types, while the older layers (L-2 and L-1) exhibit three to four distinct fiber types. The histograms and corresponding GMM estimations for all the SEM images analyzed in this study are given in Supporting Figs. S4 and S5 to provide further insights into the variation of fiber types within the mycelium network at different growth depths.

The left panel of Fig. 2 presents a collective analysis of 15 microstructures, showing the variation in dominant hyphal radii as a function of the growth depth. The plot displays the range of dominant hyphal radii, varying from the thinnest at 0.17 μm to the thickest at 1.2 μm within the mycelium network. These values are consistent with the previous studies^25,27^, which reported a distribution range of ~ 0.1–1.5 μm. In comparison, our study further resolves the discrete nature of the hyphal types and identifies network heterogeneity as a function of growth depth. Thinner hyphae with mean radii of approximately 0.17 μm and 0.34 μm are detected at each growth depth and occur more frequently (grey background) than other fiber types. Further, the frequency of their occurrence is comparable at the older growth (L-1), but the fiber radius of ~ 0.34 μm dominates the younger growth (L-2 and L-3). GMM analysis also revealed the doubling nature of the fiber radii in the network (grey background bins represents predominant radii with 0.17 μm, 0.34 μm, etc., Fig. 2) across all growth depths. A careful analysis of SEM micrographs (in supporting Figs. S6 and S7) showed the higher frequency of thinner fibers (~ 0.17 μm) for older growth networks results from the bifurcation of the leading hyphae. These observations support the growth mechanism by branch bifurcation, *i.e.*, as the radii of the dominant hyphae halved in size^36,37^. Also, a higher occurrence of thicker hyphal radii (> 0.75 μm) for older growth networks due to fiber bundling is observed.

**Figure 3:**
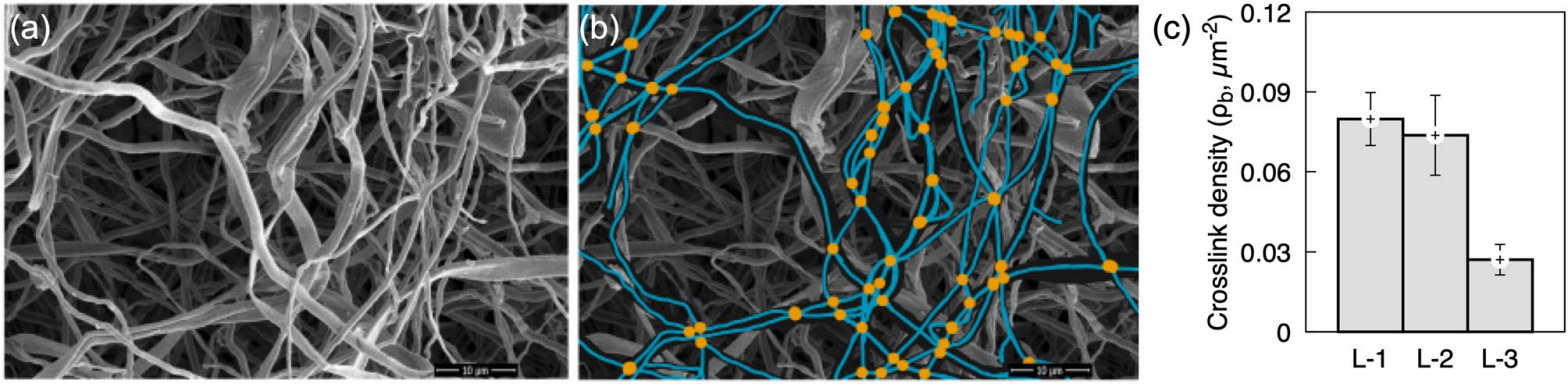
(**a**) SEM micrograph from L-1, (**b**) overlay of the manually-annotated skeleton on the SEM micrograph (blue lines) with marked intersections (yellow points) are presented. (**c**) The hyphal crosslink density (${\rho }_{b})$ as a function of mycelium growth is shown. Error bars represent the standard deviation of crosslink density across five images per depth.

*Hyphal network crosslink density*: The representative SEM micrograph (Fig. 3a) and its corresponding segmentation with intersections (crosslinks, yellow points) on the skeleton (Fig. 3b) are presented. The crosslinks in a random mycelium network originate from the branching of leading fibers, overlapping or entanglement of hyphae, and bonding between neighborhood filaments when their separation distance is within short-range interactions (generated by cell wall proteins and other biopolymers). Figure 3c illustrates the hyphal crosslink density (${\rho }_{b}$), which is quantified as the number of crosslinks per unit area of the micrograph. The crosslink density decreased by 65% along the thickness of mycelium tissue, *i.e.*, the oldest growth (L-1) having an average crosslink density of 0.079 μm^-2^ was reduced to 0.027 μm^-2^ for the youngest growth (L-3). The higher crosslink densities observed for the oldest growth correlate with the higher fiber density (and types) observed near the feed substrate (Fig. 2, L-1). In fact, the higher network density in the older growth promotes fiber proximity and a possibility of fiber fusion during the drying and baking processes (as observed in supporting information S4). This indicates that the mycelium networks consist of a combination of bonded (bifurcated) and non-bonded (interact via topological constraints) crosslinks^38^.

**Figure 4:**
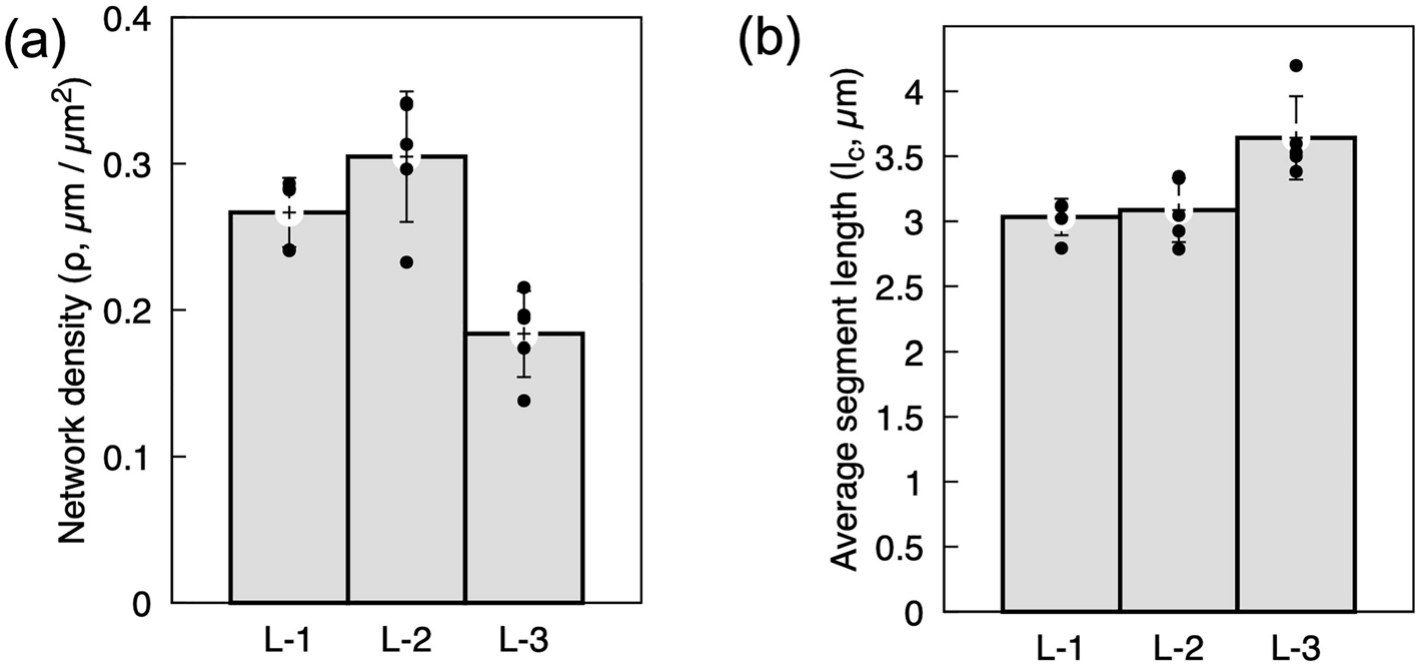
The network characteristics (**a**) network density (ρ) and (**b**) segment length (*l*_*c*_) along the growth direction are presented. The values were obtained from five micrographs per depth.

Along with the crosslink density, two other network characteristics, *i.e.*, the network density of the mycelium (*ρ*) defined as the total length of the connected skeleton per unit network volume (excluding dangling and free ends of the fibers); and the average segment length (*l*_*c*_) defined as the distance between two consecutive crosslinks along the fiber were estimated as a function of mycelium growth. The segmentation procedure employed for the pore area estimation was used for determining these parameters. The results showed the lowest network density for the younger mycelium growth (L-3) at 0.18 μm/μm^2^, while the highest density for L-2 at 0.30 μm/μm^2^. However, L-2 exhibited the highest variance in the network density (Fig. 4a), indicating that the networks become denser as the mycelium grows. Conversely, the mean segment length increased from ~ 3.03 μm at L-1 to a maximum of ~ 3.64 μm at L-3. The segment length for L-2 was observed to be similar to L-1, with no significant difference within the standard deviation in the data (Fig. 4b). This suggests that the average length of segments between the crosslinks increases as the mycelium grows, with the highest value reaching for L-3 (younger growth). To characterize mycelium, estimating network density from SEM images is a more appropriate approach at meso length scales than using the overall density of mycelium.

**Figure 5:**
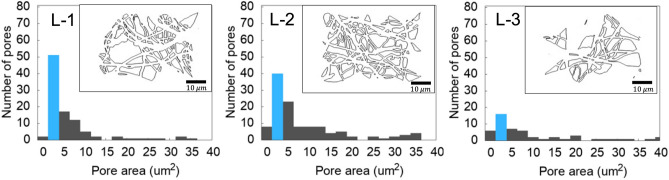
Representative histograms of pore-size distribution for the oldest (L-1), medium (L-2), and youngest (L-3) mycelium growth. The insets represent annotated skeletons that only include complete pores within the SEM micrograph.

*Pore area and Porosity*: Figure 5 presents the histograms of the pore areas measured for different growth depths, ranging from the oldest (L-1) to the youngest (L-3) growth. The insets within the figure depict the identified complete pores within the corresponding SEM micrograph used to generate the pore area histograms. The analysis of histograms demonstrates that pores with an area of ~ 2.75 μm^2^ are the dominant pore size across all growth depths (blue bar). Further, the histograms for younger mycelium tissue (L-3) show a wider distribution of pore sizes compared to the older growth tissues (L-1 and L-2).

Table 1 summarizes the total number of full pores*,* the fraction of dominant pores (~ 2.75 μm^2^, the dominant bin in the histograms), small pores (< 5.5 μm^2^, the first three bins in the histograms), and the network porosity along the mycelium growth direction. The averaged values and the vector of five values extracted from different micrographs obtained at each depth are presented. A significantly higher number of full pores were identified for the older growth tissues (L-1, L-2) compared to the younger growth (L-3). Additionally, the fraction of dominant pores with an area of approximately 2.75 μm^2^ was found to be ~ 32% for L-3, while it increased to 44 and 50% for L-1 and L-2, respectively. Similarly, the fraction of small pores (pore area < 5.5 μm^2^) was significantly lower for the younger growth (56% for L-3) compared to the older growth (67% for L-1 and 71% for L-2). This behavior is consistent with the crosslink density calculations, where the older growths presented a higher number of intersections per unit area (Fig. 3).

**Table 1:** A summary of the pore characteristics of the random fibrous network of mycelium along its growth direction.

	L-1	L-2	L-3
Total number of identified full pores	[78, 102, 103, 101, 111] 99	[72, 120, 154, 133, 103] 116.8	[40, 38, 56, 66, 59] 51.8
Fraction of dominant pores (2.75 μm^2^)	[0.49, 0.58, 0.45, 0.52, 0.48] 0.50	[0.42, 0.34, 0.53, 0.49, 0.43] 0.44	[0.32, 0.27, 0.31, 0.47, 0.24] 0.32
Fraction of small pores (area < 5.5 μm^2^)	[0.72, 0.78, 0.67, 0.71, 0.68] 0.71	[0.59, 0.60, 0.82, 0.71, 0.60] 0.67	[0.59, 0.42, 0.56, 0.63, 0.59] 0.56
Porosity	[0.52, 0.50, 0.47, 0.50, 0.61] 0.52	[0.60, 0.46, 0.48, 0.53, 0.52] 0.52	[0.68, 0.65, 0.63, 0.61, 0.66] 0.65

Table 1 also summarizes the porosity of the mycelium network as a function of the growth depth. While there was an insignificant change in porosity for older growth (L-1 and L-2), the porosity increased by 65% for the younger growth tissue (L-3). The mycelium displayed a sparsely porous structure similar to natural random fibrous materials such as fibrous wood, which typically ranges between 0.1 and 0.6^39^. Further, it is worth noting that the estimation of the true porosity of the three-dimensional heterogeneous network using two-dimensional SEM micrographs may not be accurate. This issue is currently being addressed by imaging the dried mycelium using Computer Tomography scanning methods, although this analysis is beyond the scope of the current manuscript.

### Growth-dependent mechanical properties of intentionally-grown dry mycelium

**Figure 6:**
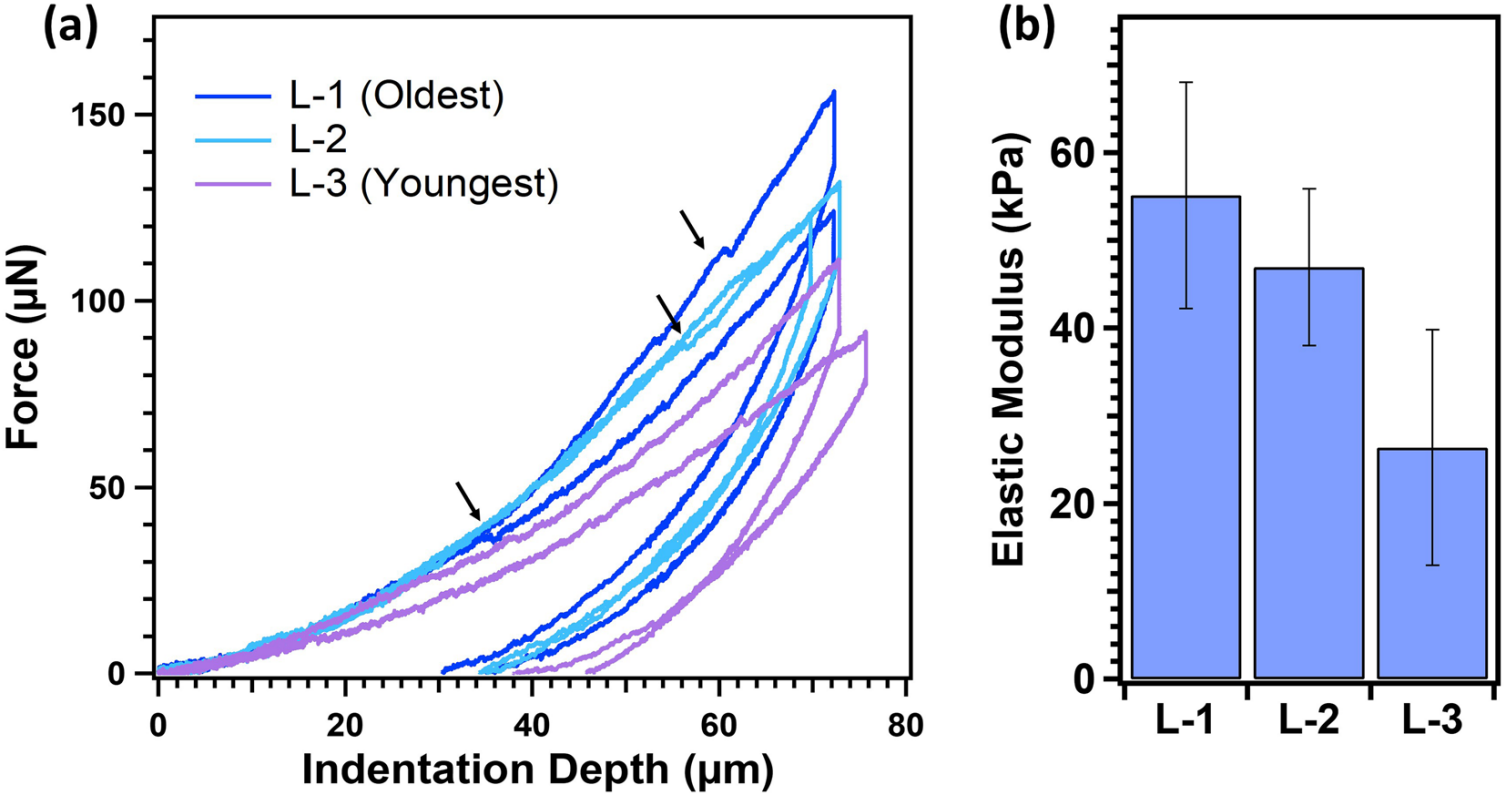
(**a**) Representative force vs. indentation depth (*F-d*) curves measured along the growth direction of the dried mycelium network, *i.e.*, from L-1 (oldest) to L-3 (youngest), and (**b**) the variation in the elastic moduli (kPa) as a function of mycelium growth depth (across 30 mm), as estimated from Hertz model, is presented.

Figure 6a presents representative *F-d* measurements obtained on mycelium sections from different growth depths using a microindentor with a radius (*R*) of ~ 92 μm. The maximum indentation depth (*d*_*max*_) was maintained at ~ 65–75 μm, which is significantly smaller than the thickness of the mycelium sample (*t*_*f*_ ~ 10 mm). This results in *d*_*max*_*/t*_*f*_ < *0.1* minimizing any potential substrate effects during the *F-d* measurements. It should be noted that a constant indentation depth was not always achieved due to the inherent heterogeneity at the mycelium interface. Additionally, the surface roughness of the mycelium sections can also influence the mechanical measurements. Estimating the actual roughness of the mycelium section is challenging due to the presence of dangling fibers at the interface. However, the effects of surface roughness are minimized in these measurements by employing probe radius and indention depths significantly larger than the dominant pore size of the mycelium network (~ 2.75 μm, as described in Table 1).

*Indentation analysis*: An appropriate contact mechanical model is necessary to estimate the elastic (Young’s) modulus of the mycelium network from the measured *F-d* curves. The indentation speed was held < 3 μm/s to generate a quasi-static elastic deformation of the network. In spite, during the indentation process, a significant number of loading curves displayed pop-in events at higher loads (black arrows in Fig. 6a). These events suggest slips or instabilities of the probe arise from the non-recoverable reorganization of the network beneath the indentation probe. As a result, applying the Oliver-Pharr model^40^, commonly used for elastic modulus calculations, to the initial 20% of the retraction curves lead to inaccurate results.

Instead, Hertz's contact mechanical model, defined for small deformations of linearly elastic materials at the contact region formed between the probe and the material, was explored on the approach curves^41^. Despite its limitations, the Hertz model has been cautiously employed to measure the mechanical properties of soft matter^42–45^ and other network structures^44^ that exhibit high deformations and/or non-linear behavior. The strength and mechanical behavior of the random fiber networks are shown to be influenced by the network characteristics^46^. A recent study by Merson et al*.*^47^ on the mechanical deformation of fibrous networks showed negligible network densification under the indenter when the probe radius was significantly larger than the network mesh size, *i.e.,* $$R/{l}_{c}$$ > 12. Such cases followed an elastic deformation of the network and validated the applicability of the Hertzian model in the analysis of the mechanical behavior of fibrous networks. In fact, the structural analysis of the mycelium network in this study measured network segment lengths (*l*_*c*_) ~ 3.0–3.5 µm (Fig. 4b), resulting in network mesh size ($${R/l}_{c})$$ under indentation ranging between 26.2 and 30.6 [−]. These values are significantly > 12, justifying the employment of the Hertzian model for the deformation of mycelium networks using microindentation technique^47^.

In the indentation analysis, the Hertz contact model relates the applied force (F) to the indentation depth (d) through $F= 4/3{E}^{*}{R}^{0.5}{d}^{1.5}$, where R is the indenter radius and E* is the reduced modulus. Assuming an infinite rigidity to the indenter probe, the reduced modulus is calculated as 1/E* = (1-υ_sample_^2^)/E, where E, υ represents the elastic modulus and Poisson's ratio of the sample, respectively. The accuracy of the Hertz fit largely depends on the appropriate recognition of the initial point of contact and avoiding fits to non-linear deformations at higher contact forces. As the probe approaches the mycelium network, the initial deformation is dominated by the compression of dangling hyphae at the surface. To avoid the uncertainty present during the initial deformation, the approach proposed by Garcia et al.^48^ is employed. This approach utilizes the d*F*/d*u* vs. F relationship according to the equation $dF/du=K.{F}^{n}$ (a derivative of the Hertz model, supporting information 5). This approach avoids the need to identify the precise initial point of contact and enables the use of an arbitrary depth value larger than the noise in the data during the initial approach. The representative plots are presented in the supporting Fig. S8, and Hertz fits employed to indentation forces ranged between 5 and 30 μN. Figure 6b presents the average elastic moduli (E) obtained from the Hertz fits for different growth depths. The oldest growth (L-1) exhibits an average elastic modulus of 55.1 ± 12.9 kPa, which systematically decreased to 26.5 ± 13.4 kPa for the youngest growth (L-3). This corresponds to an approximately 52% decrease in elastic modulus across the 30 mm-thick mycelium tissue.

**Figure 7:**
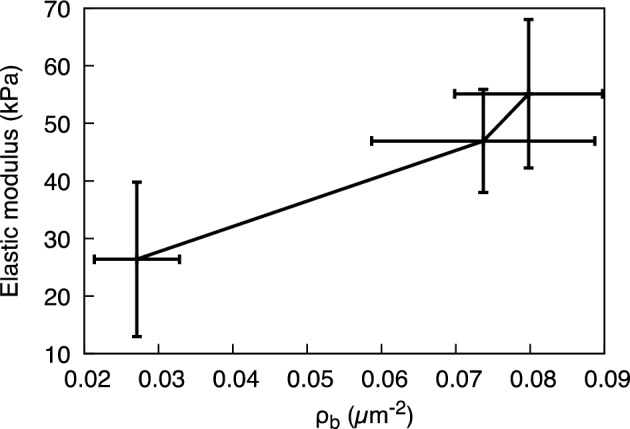
Structure–property relationship for the mycelium: Elastic modulus of the local network as a function of its crosslink density, ${\rho }_{b}$. Higher ${\rho }_{b}$ represents older growth (L-1).

A correlative analysis between the measured mechanical modulus of the mycelium network and various structural parameters extracted from the networks, as a function of mycelium growth, reveals a direct relationship between the mechanical modulus and the crosslink density (${\rho }_{b})$ of the network even at meso length scales(Fig. 7). The older-growth network, which presented the highest crosslink density, measured the highest elastic modulus. Previous studies on random fibrous networks under uniaxial stress (macroscale loading) also demonstrated a similar dependence of network modulus with network density^27^. In our specific case, the mycelium network exhibits a range of hyphal radii (*r*) from 0.2 to 1.2 µm (Fig. 2) and network segment length ranging between 3.0 and 3.5 µm (Fig. 4b) across all depths, resulting in *r/l*_*c*_ values ranging from 0.06 to 0.40. The high *r/l*_*c*_ render *athermal* (insensitivity to thermal fluctuations) nature to mycelium network, where the mechanical deformations and strain energy are transmitted through forces and moments of inertia at the fiber intersections^49^. This leads to the observed direct correlation between the stiffness of the *athermal* network and the crosslink density of the network (bearing area, Fig. 7), as also previously shown^46,49^.

Furthermore, our studies have also revealed a direct relation between the crosslink density and the segment length, expressed as ${\rho }_{b}\sim 1/{l}_{c}$, and thence to elastic modulus (Fig. S9)^50,51^. This study indicates that the macroscopic deformation of the mycelium network, as measured by uniaxial testing equipment, is translated to mesoscale^46^. This behavior, observed for the first time in the mycelium network, shares similarities with the affine behavior observed for simple elastic networks and non-linear elastic biological networks^52,53^.

## Conclusions

In summary, advanced algorithms employed for microstructural characterization reveals a gradient in the structural properties of mycelium as growth progresses intentionally. The oldest growth network (L-1) exhibits multiple dominant hyphal radii, higher intersection densities, and smaller pores compared to the younger growth network. The elastic moduli measured through micro-indentation mirrored this trend. A variation of approximately 30 kPa in mechanical modulus is observed over a 30 mm vertical height of the mycelium tissue, with the oldest growth network presenting the highest elastic modulus. This indicates a subtle but clear transition in structural and mechanical properties along the growth direction. Future studies will focus on exploring variations in growth conditions and feed parameters to generate tunable mycelium gradients with desired structural and functional properties.

Fungal biomanufacturing plays a crucial role in achieving various sustainability goals set by the United Nations^54,55^. To fully utilize mycelium as a sustainable material in diverse applications, targeted morphological engineering is essential. This involves understanding the mechanistic factors that control and influence fungal growth dynamics, including genetics, chemical and mechanical stresses, and bioreactor design. Furthermore, it is important to develop tools and methodologies that can link the heterogeneous structure (growth) of the mycelium to its mechanical and rheological properties. This is critical for identifying the design rules in morphology engineering. Our study presents a comprehensive mesoscale characterization of the structure–mechanical property of mycelium to demonstrate the impact of morphology on the mechanical properties of the mycelium. We anticipate further advancements in designing tunable gradient structures using mycelium-based constructs will result in enhanced and transformative properties, benefitting both their current and future applications.

### Supplementary Information

Supplementary Information.

## Data Availability

The datasets generated during and/or analyzed during the current study are available from the corresponding authors upon reasonable request.
