# Supplementary material for: Gradient porous structures of mycelium: a quantitative structure–mechanical property analysis
Source: Sci Rep. 2023 Nov 7;13:19285. doi: 10.1038/s41598-023-45842-5 (PMC10630317; doi:10.1038/s41598-023-45842-5)
Supplement: Supplementary file 1 — Supplementary Information. [file 41598_2023_45842_MOESM1_ESM.docx]

**Supporting Information for**

**Gradient Porous Structures of Mycelium: A Quantitative Structure-Mechanical Property Analysis**

Eric Olivero^1^, Elzbieta Gawronska^2^, Praveena Manimuda^3^, Devyani Jivani^1^, Faemia Zullfikar Chaggan^1^, Zachary Corey^1^, Thaicia Stona de Almeida^1^, JessieBie-Kaplan^4^, Gavin McIntyre^4^, Olga Wodo^1,*^ and Prathima C. Nalam^1,*^

***^1^*** *Department of Materials Design and Innovation, University at Buffalo, Buffalo, NY 14226, USA****^2^*** *Faculty of Mechanical Engineering and Computer Science, Czestochowa University of Technology, Czestochowa, 42201, Poland*

***^3^*** *Horiba Instruments Inc, Piscataway, NJ 08854, USA*

***^4^*** *Ecovative Design, LLC 60 Cohoes Ave, Green Island, NY 12183, USA*

**Corresponding Authors:* [*prathima@buffalo.edu*](mailto:prathima@buffalo.edu)*,* [*olgawodo@buffalo.edu*](mailto:olgawodo@buffalo.edu)

**Supporting Information 1 (S1):** Impact of magnification of SEM images on the data resolution of extracted descriptors.

The below images are acquired at four different magnifications, i.e., 800x, 1Kx, 3Kx, and 15Kx. With the increase in image magnification, the data resolution, *i.e.*, the frequency and density of the bins in the histogram, increased, leading to a better description of the dominating fiber radii. However, due to the limited number of fibers per image, the extraction of statistically significant values of microstructural descriptors could not be achieved. Hence, the intermediate magnification of 3Kx has been selected as one offering statistical significance balanced with the good resolution of individual fibers.

**
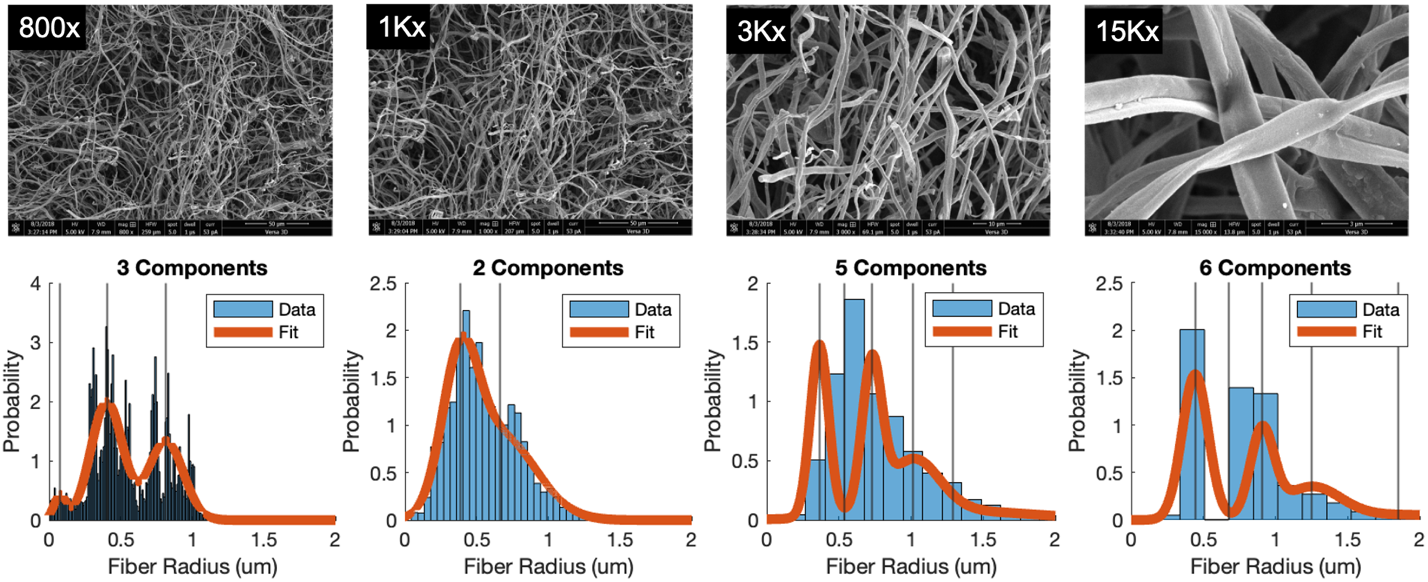
**

**Figure S1:** Representative SEM images of the mycelium network acquired at different magnifications and the corresponding distribution of the fiber radius extracted from the images are presented.

**Supporting Information 2 (S2):** Procedure for segmentation and noise reduction of SEM images for fiber extraction.

Representative raw and segmented images (using DiameterJ) are presented. The original 8-bit grayscale image is first converted into a segmented image where white pixels correspond to the fibers, and the black pixels represent the voids. Segmentation includes four steps: (a) Statistical region merging to homogenize pixel intensity and reduce noise before thresholding, (b) Otsu thresholding to generate binary images, and (c) noise reduction. Due to the nature of the acquired images (charging effects, non-homogeneous networks), the thresholding process creates noise in the image. Hence, a 3x3 median filter is passed over the image to reduce unidentified pixels generated during thresholding. Morphological erosion and dilation operations are performed to remove fiber edge discontinuities. Finally, (d) visual comparison with manual editing is employed to further improve the accuracy of the segmentation.


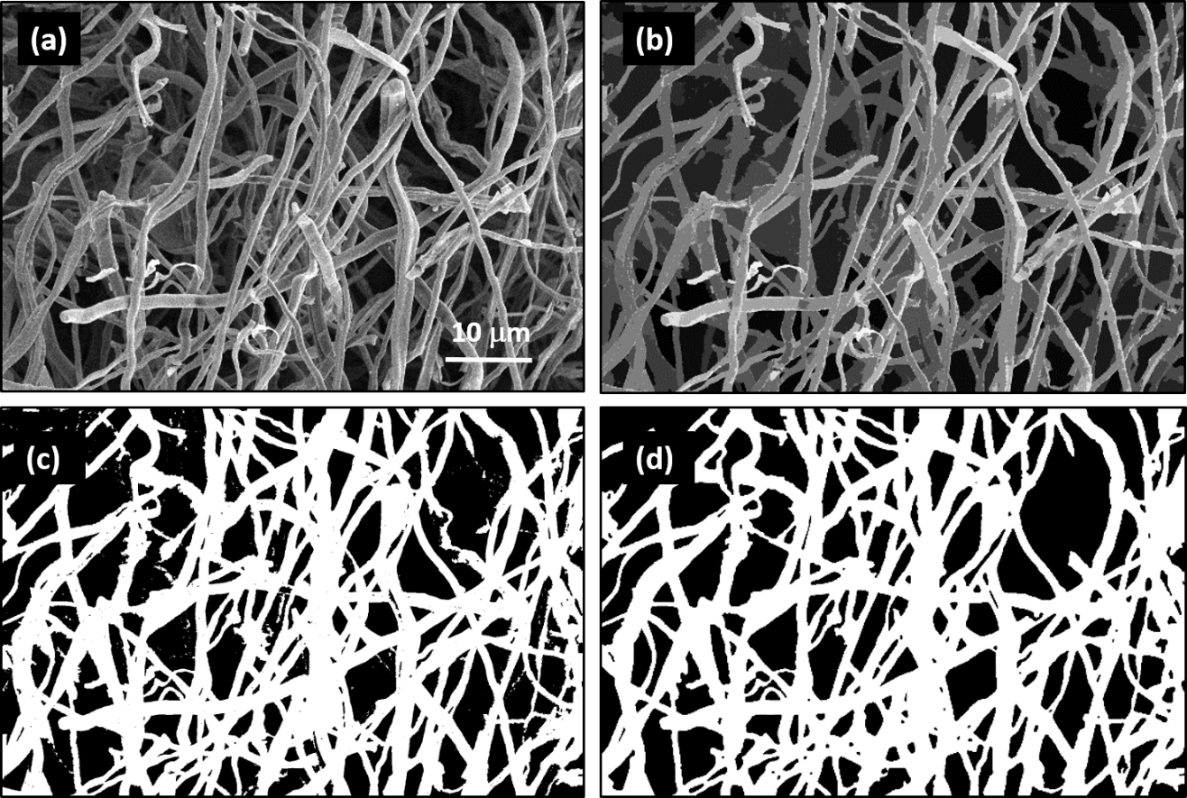


**Figure S2:** Representative raw and segmented images (using DiameterJ) of SEM micrograph (3kX) are presented.

**Supporting Information 3 (S3):** Estimation and analysis of the fiber radii.

To deconvolute the contribution of each type to the histogram Gaussian mixture model (GMM) was used on generated histograms. Below we provide details on the histogram generation and GMM modeling. The complete set of fifteen histograms with GMM fitted is also included at the end of this section.

*Generating distributed histograms for fiber radii*: The figure below depicts the basic steps involved in the radii histogram generation: The segmented images (a) are employed to extract a distance transformation of the segmented image, which measures Euclidean distance from each pixel in the fiber to its nearest orthogonal background pixel. (b) The segmented images are skeletonized by recursively eroding the edges of the fibers until a common central line is met. Overlaying these two transformations, the Euclidean distance between the centerline of the fiber and the nearest orthogonal pore is determined. This value is extracted for each centerline pixel and is considered as the fiber’s radius. (c) The intersections of fibers cause an increase in the actual radius of the fiber; hence the skeletonized image and distance transformation are overlaid, and the radius value surrounding the intersection is avoided. (d) The extracted fiber radii are plotted as histograms for further analysis.


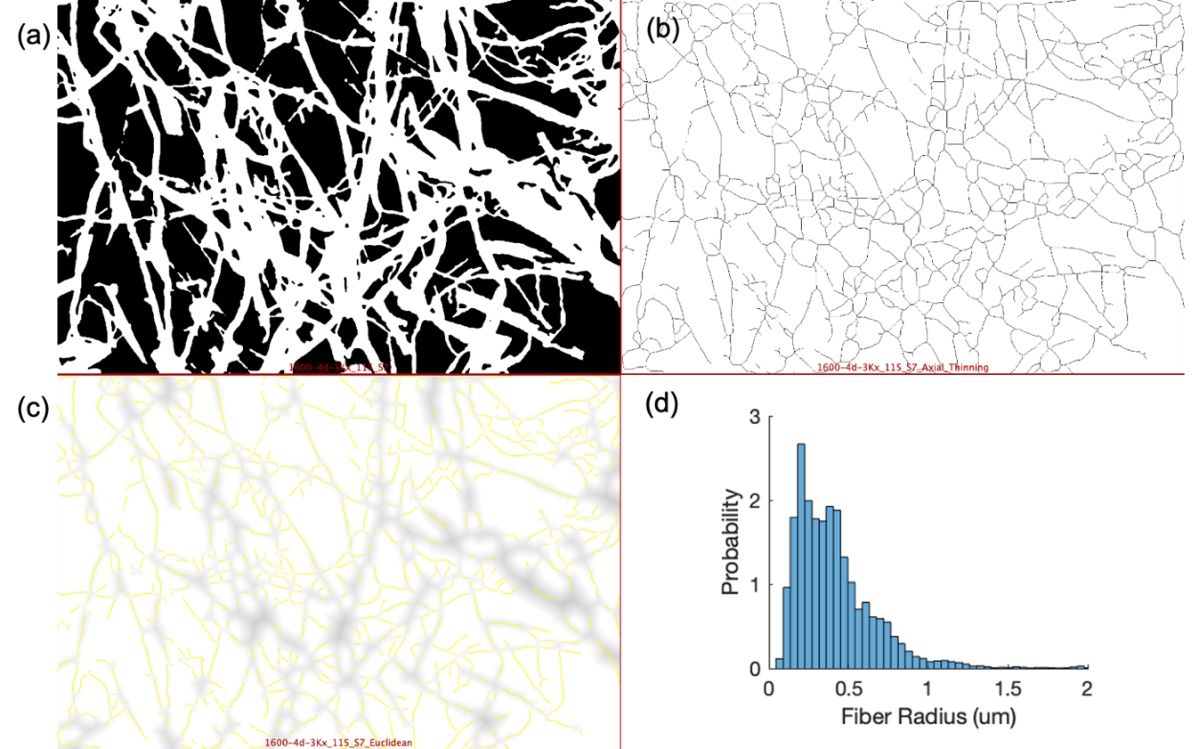


**Figure S3:** Step-by-step procedure to extract the radii histogram from the segmented SEM image (using DiameterJ).

*Gaussian Mixture Modeling:* The heterogeneous mycelium network, due to fiber age, type of fiber, anagenesis, etc., results in fibers of various radii. To extract the predominant fiber types from the distributed histogram, a GMM is employed. This model assumes that the radius of the leading fibers is distributed normally around a central value with some variance. The GMM is the sum of Gaussian fits, each having a distinct mean, variance, and mixing parameter. The GMM additionally requires a predetermined number of components for appropriate fit. While higher components improve the fit quality but can lead to an overfit and obscure the generalizations of the results.

Hence, while employing GMM to the distribution, the expectation-maximization method was employed where the expected value of the likelihood function is maximized. The Akaike information criterion (AIC) is employed to determine the optimal number of model components and is presented as,

$$\boldsymbol{AIC=}\mathbf{2} \boldsymbol{k-2}\ln\left( \boldsymbol{L} \right)$$

where *k* is the number of independent variables used, and L is the log-likelihood estimate. Here, *k = 3n* represents three parameters: mean, standard deviation, and mixing factor for each of the *n* Gaussians. The value of the AIC decreases with an increase in the likelihood value (*L*). The higher values of *n* reward an accurate model but can also result in overfitting of the histogram with each added component*.* In this study, the AIC values are calculated up to six components. As the AIC reaches a minimum value, adding more components does not sufficiently improve the model and is considered the optimal number of components. This is referred to as the “elbow rule”, where the “pivot” point in the elbow is the optimal model.


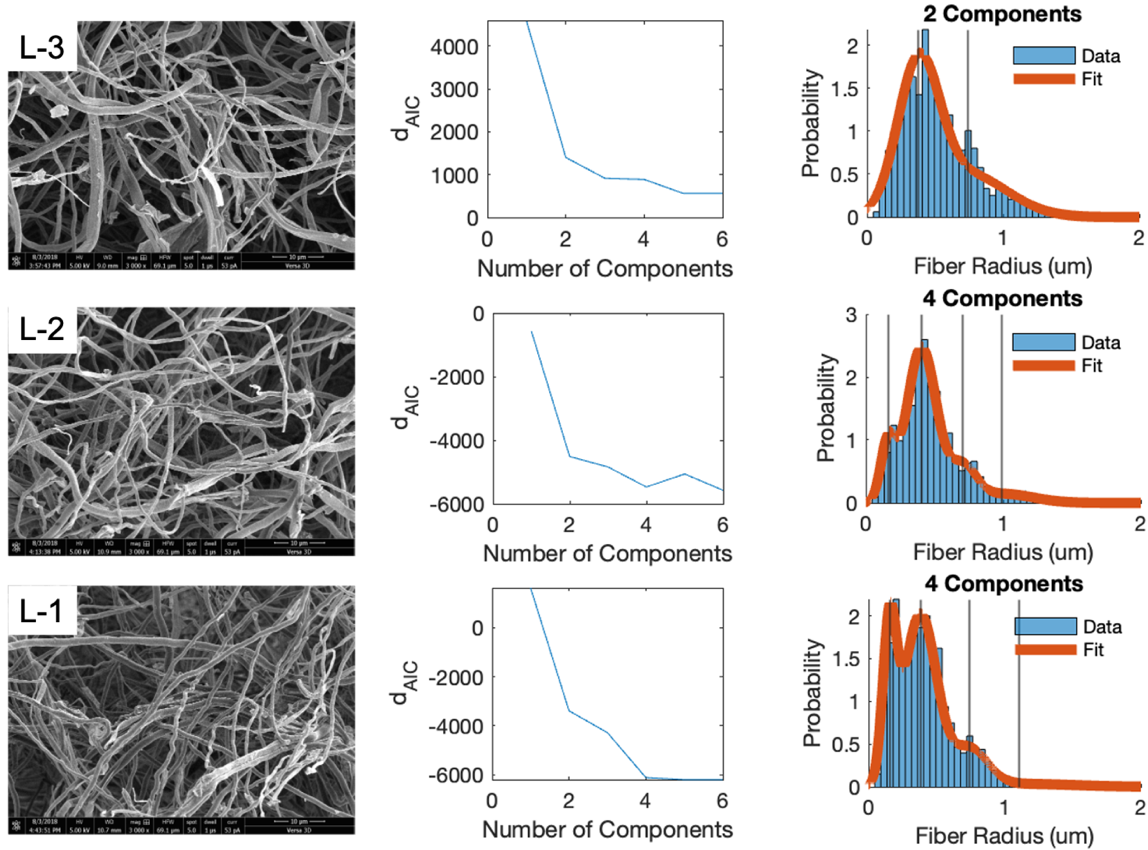


**Figure S4:** Representative SEM micrographs, Akaike Information Criterion (AIC), and fibrous radius histogram with the GMM fit (orange line) as a function of mycelium growth.


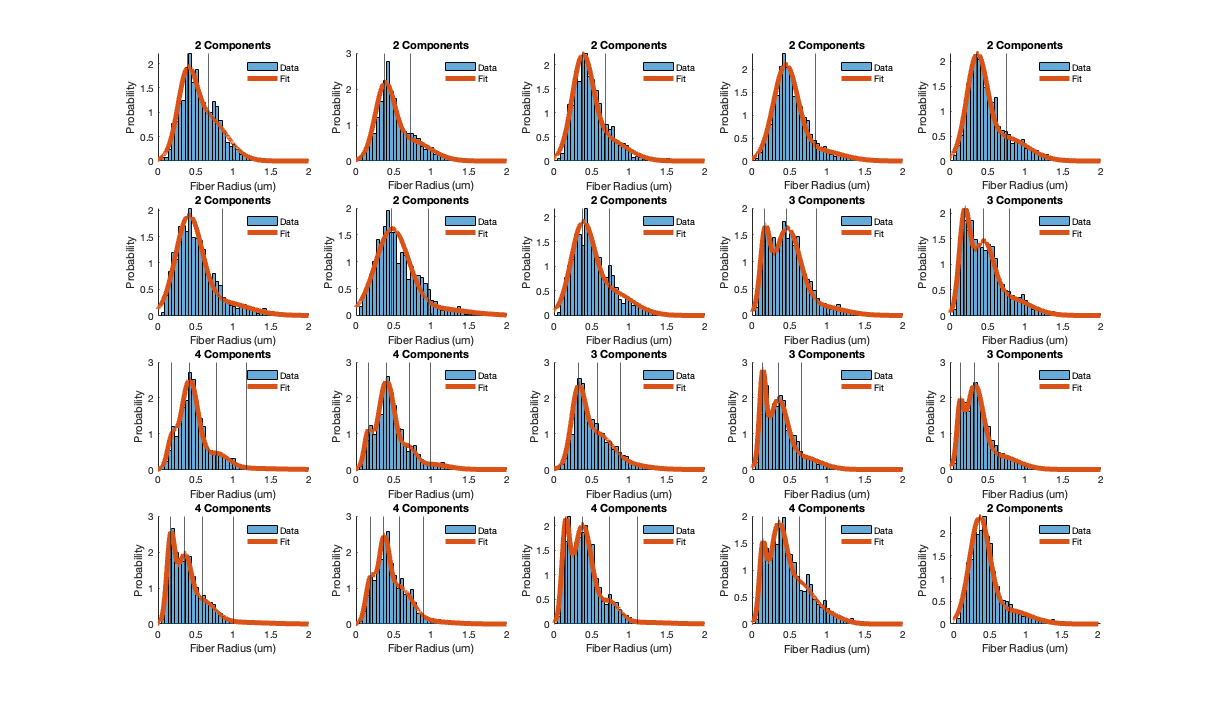


L1

L2

L3

**Figure S5:** The histograms and the corresponding GMM-fits based on the number of components (*n*) as estimated by the AIC model are shown for all the 15 SEM micrographs (5 per depth) used in this study.

**Supporting Information 4 (S4):** Interpretation of the Mycelium fiber radii analysis

Representative SEM micrographs and the corresponding segmented images for younger and older mycelium networks are presented. Fiber segmentation algorithms described in Supporting Information 2 were employed to generate segmented images.

In Figure (a), the segmented images for the older growth networks showed significantly higher thinner fibers (< 0.2 μm, red line in segmented images) compared to younger growth mycelium tissue. The identified thin fibers seem to originate from the branching of the leading mycelium hyphae (red boxes in the SEM images).

**
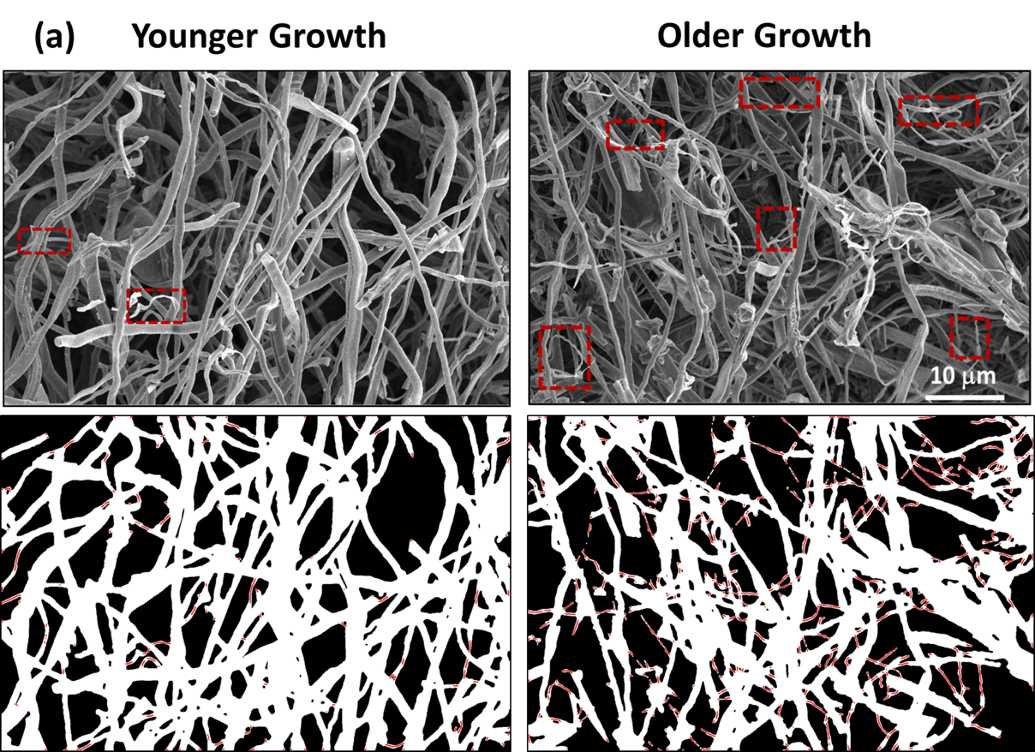
**

**Figure S6:** SEM micrographs for younger (L-3) and older (L-1) growth mycelium indicating thinner fibers in the networks.

Figure (b) identifies a higher frequency of thicker fiber for the older growth networks (red boxes). A careful look into the SEM images identifies the thicker fibers as bundles of two or more hyphae. Especially when the mycelium networks are denser (older growth), the proximity of the fibers can result in hyphal fusion during the drying and baking processes.


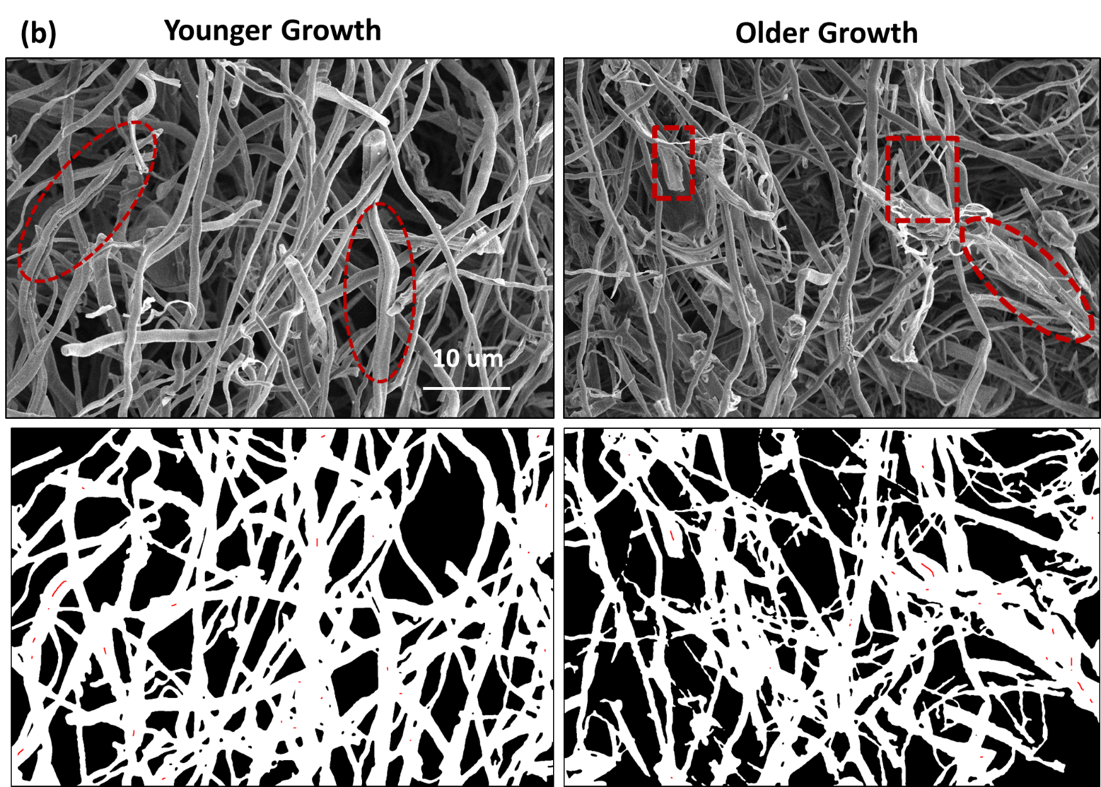


**Figure S7:** SEM micrographs for younger (L-3) and older (L-1) growth mycelium indicating thicker fibers in the networks.

**Supporting Information 5 (S5):** Estimation of elastic modulus of the mycelium network using Hertzian contact mechanical model.

The Hertz fits to *dF/du* vs. *F* is plotted as a function of mycelium growth, with the oldest growth (L-1, blue), the medium growth (L-2, Green), and the youngest growth (L-3, red) presented.

The Hertz equations used for the fit are described below:

$$F= \frac{4}{3} E^{*}R^{0.5}d^{1.5}$$

$\frac{dF}{du}=K.F^{n}$ with $K=P* k^{1/P}$ --- *eq. 1*

where *P* =1.5, *n* = 1/3 and$k=\left( \frac{4}{3} \right)E^{*}R^{0.5}$

The reduced modulus is estimated by obtaining the least-square error fit to the power law model (Equation 1), and E* is estimated from the fit output (*K*). Subsequently, the elastic modulus of the mycelium network (E) is obtained from E*. The values for Poisson ratio = 0.3 [-] and radius R = 92 μm were employed in this study [1].

Hertz fits in the *dF/du vs. F* curves were restricted to indentation forces between 5 to 30 μN. At forces higher than 30μN, the occurrence of pop-in events is often observed, resulting in changes in the indentation slope. Hence, the fits to higher forces were avoided in this study.

**
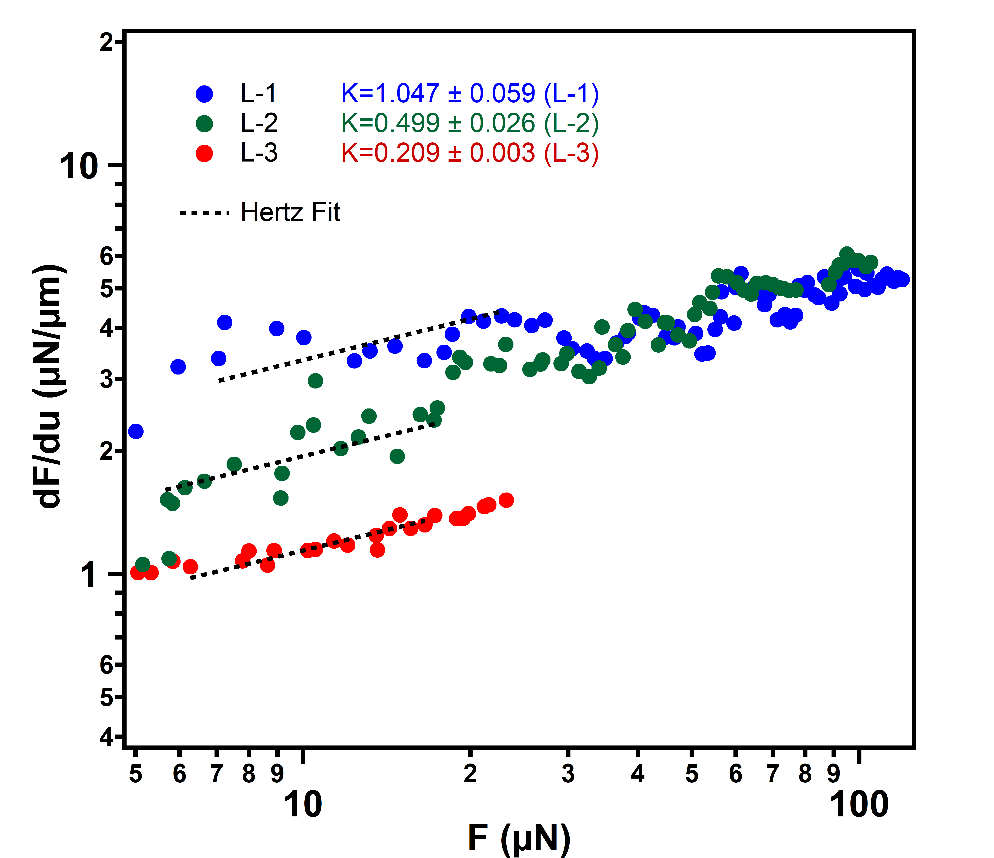
**

**Figure S8:** Representative *dF/du* vs. *F* as a function of mycelium growth (L-1, older growth and L-3, Younger Growth) as extracted from the microindentation measurements are shown. Fits to the Hertz model are presented.

**Supporting Information 6 (S6):** Establishing structure-property relations of mycelium network.

A direct correlation between the crosslink density with the inverse segment length of the network is observed, as observed for the fibrous networks [2]. Higher elastic modulus is measured for older growth mycelium that showed the highest crosslink density or the lowest segment lengths.

**
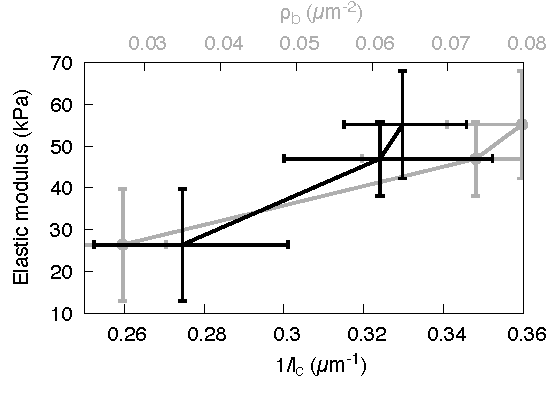
**

**Figure S9:** Structure-property relationship for the mycelium: Elastic modulus of the local network as a function of its crosslink density, $\rho_{b}$ , and inverse of segment length (1/*l_c_*). Higher $\rho_{b}$represents older growth (L-1).

**References:**

**[1]** Merson, J., Parvez, N. & Picu, R. C. Probing soft fibrous materials by indentation. *Acta Biomater.* **163**, 25–34 (2023).

**[2]** Islam, M. R., Tudryn, G., Bucinell, R., Schadler, L. & Picu, R. C. Morphology and mechanics of fungal mycelium. *Sci. Rep.* **7**, 13070 (2017).
